# Supplementary material for: Global warming accelerates soil heterotrophic respiration
Source: Nat Commun. 2023 Jun 10;14:3452. doi: 10.1038/s41467-023-38981-w (PMC10257684; doi:10.1038/s41467-023-38981-w)
Supplement: Supplementary file 1 — Supporting Information [file 41467_2023_38981_MOESM1_ESM.pdf]

Supplementary Information:

Global warming accelerates soil heterotrophic respiration

Alon Nissan[1*], Uria Alcolombri[1], Nadav Peleg[2], Nir Galili[3], Joaquin Jimenez-Martinez[1,4], Peter Molnar[1] and Markus Holzner[4,5]

[1]Institute of Environmental Engineering, Department of Civil, Environmental and Geomatic Engineering, ETH Zürich, Zürich, 8093, Switzerland.
[2]Institute of Earth Surface Dynamics, University of Lausanne, Lausanne, 1015, Switzerland.
[3]Geological Institute, Department of Earth Sciences, ETH Zürich, Zürich, 8092, Switzerland.
[4]Department of Water Resources and Drinking Water, Swiss Federal Institute of Aquatic Science and Technology, EAWAG, Dübendorf, 8600, Switzerland.
[5]Biodiversity and Conservation Biology, Swiss Federal Institute for Forest Snow and Landscape Research, WSL, Birmensdorf, 8903, Switzerland.

*Corresponding author(s). E-mail(s): anissan@ethz.ch;

Supplementary Figures and Tables

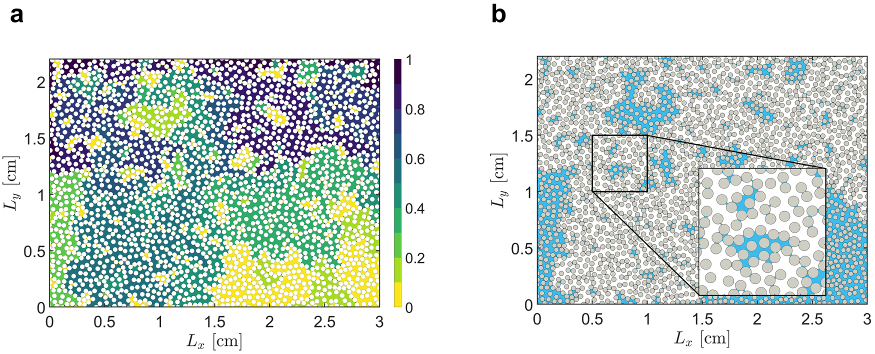

Supplementary Fig.. 1: **(a)** Example of a single realization of the invasion–percolation algorithm (grain size, $\lambda_c = 0.2$ mm). The colour bar represents the soil saturation degree ($\tilde{\theta}$) when each water cluster was invaded; blue are the first and yellow are the last invaded clusters. **(b)** Air (white) and water (blue) distribution in the soil matrix (soil grains in grey) for $\tilde{\theta} = 0.4$ from the same run of the invasion–percolation algorithm as in (a). The magnified inset shows the formation of small water patches, which are held by capillary forces.

Supplementary Table 1: Parameters for the HR Model and Invasion-Percolation Simulations

Parameter	Units	Description
D_m	$m^2\ s^{-1}$	Oxygen diffusion Coefficient
C_{O_2}	mol m^{-3}	Oxygen concentration
C_s	mol m^{-3}	Substrate (dissolved organic carbon) concentration
V_m	mol $m^{-3}\ s^{-1}$	Maximum reaction rate
$K_m(s)$	mol m^{-3}	Michaelis constant for dissolved organic carbon
$K_m(O_2)$	mol m^{-3}	Michaelis constant for dissolved oxygen
ϕ		Porosity
λ	m	Characteristic grain size
S_c		Characteristic length of the water patches in the domain
N_c		Number of the water patches in the domain

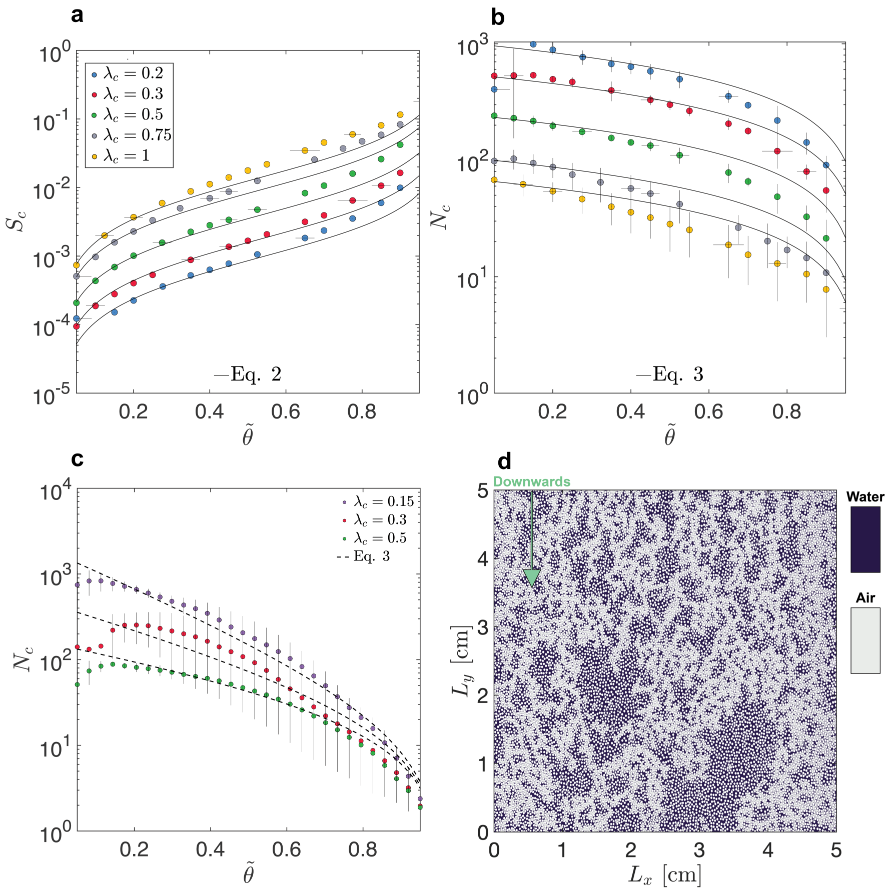

Supplementary Fig.. 2: **(a)** Number and **(b)** characteristic size of water patches from the pore-scale simulations (Fig. 2a) for different grain sizes (legend). The solid lines in a and b represent Eq. 2 and Eq. 3, respectively (see main text). **(c)** Experimental measures of drainage in microfluidic chips of different grain sizes (legend) and the comparison with the scaling law of Eq. 3 (dashed lines). The vertical error bars show the standard deviation between three replicates. **(d)** A snapshot from the drainage process inside the microfluidic chip, at $\tilde{\theta} = 0.5$ ($\lambda_c = 0.15$ mm); water in blue and air in grey. Based on these images, we extracted the water patch properties (i.e., S_c and N_c).

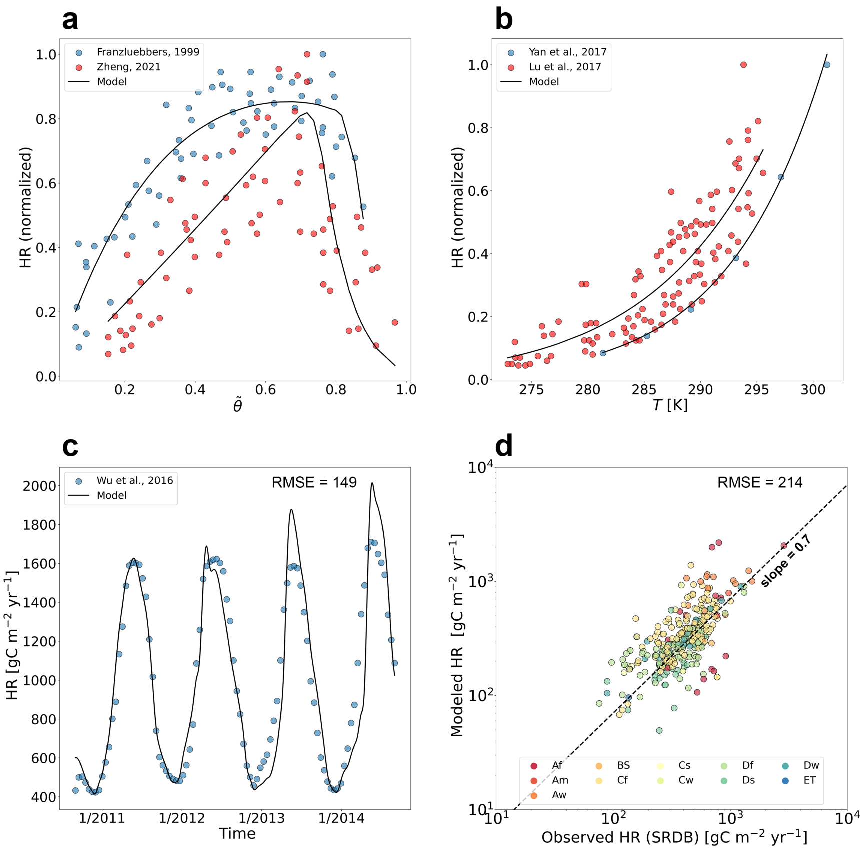

Supplementary Fig.. 3: Observations (dots) of soil heterotrophic respiration (HR) as a function of **(a)** soil saturation [1, 2] and **(b)** ambient temperature [3, 4] conditions, and the predictions of our model (solid lines), based on the reported soil texture, saturation degree and ambient temperature; dissolved organic carbon concentration was assumed to be at saturated conditions (i.e., $\frac{C_s}{C_s+K_{m(s)}} \to 1$ in Eq. 5, Methods). **(c)** A time series of soil heterotrophic respiration observations in the subtropical forest [5] were compared to model predictions. The model was parameterized using measured soil temperature, moisture, and reported soil texture. **(d)** Model predictions plotted against field observations of mean soil heterotrophic respiration (SRDB V5, [6]), at different climatic locations (colour bar indicates the Köppen climate classification of the different sites). The solid dashed line represents the strongest correlation (with a slope of 0.7) between the observed data and the model predictions. This correlation implies that the top layer of soil accounts for approximately 70% of the total heterotrophic respiration from the soil profile.

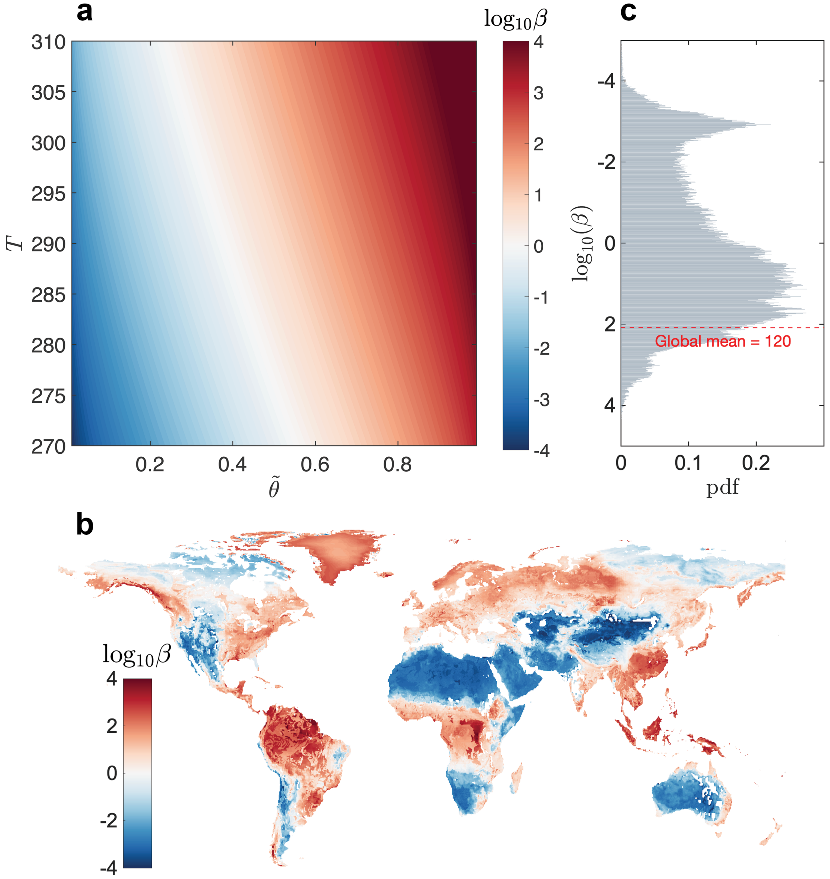

Supplementary Fig.. 4: **(a)** Two-dimensional contour surface (colour scale) based on the model (Methods Eqs. 1 - 3) of $\log_{10}(\beta)$ as a function of the surface temperature (T) and soil saturation ($\tilde{\theta}$). Note that for this particular example, the global mean grain size (0.3 mm) was used to parameterize λ_c, and dissolved organic carbon concentration was assumed to be at saturated conditions (i.e., $\frac{C_s}{C_s+K_{m(s)}} \rightarrow 1$ in Eq. 5, Methods). **(b)** Global map of the annual mean β values (year 2021); colour bar on logarithmic scale ($\log_{10}(\beta)$). **(c)** Probability density function of logarithmic β values based on the global map in **b**; the overall mean value of β is 120 (dashed red line).

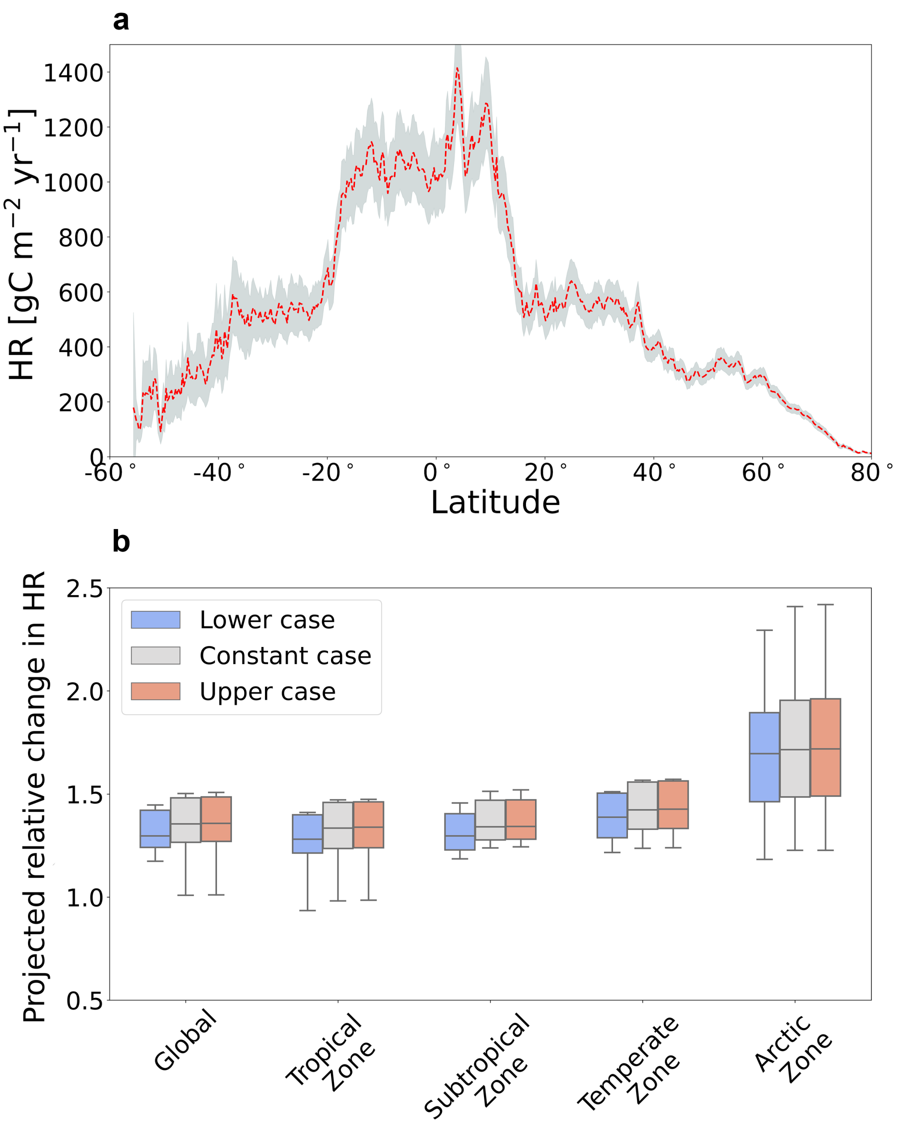

Supplementary Fig.. 5: Model Sensitivity: **(a)** 2000 Monte Carlo simulations were carried out with daily global soil temperature and moisture datasets for 2021 [7]. The red dashed line represents the relationship between annual heterotrophic respiration (HR) and latitude, based on monthly intervals as reported in the paper. The gray cloud illustrates the range of simulations based on daily distributions. The Tropical zone exhibits the highest standard deviation of about ± 20 gC m^{-2} yr^{-1}, while the largest relative uncertainty of 5% is observed around the South Pole. **(b)** Impact of changes in dissolved organic carbon concentration on heterotrophic respiration predictions until the end of the century; blue: a linear decrease of 5%, gray: constant, and red: a linear increase of 20% [8].

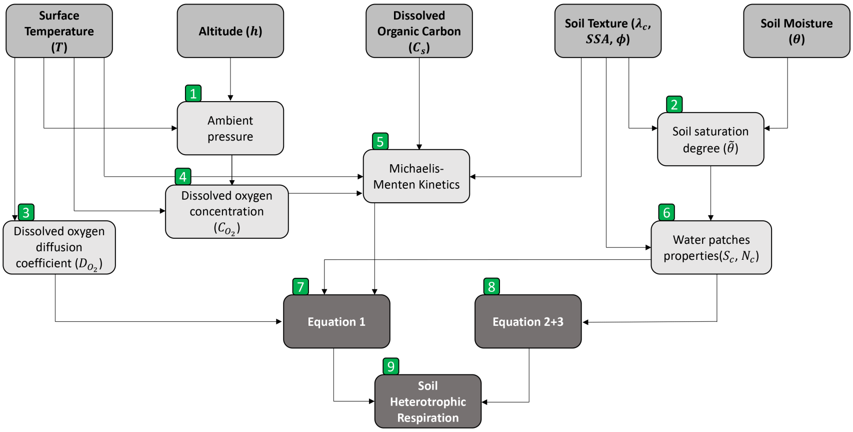

Supplementary Fig.. 6:
Schematic illustration of the model methodology to derive estimates of heterotrophic respiration from climatic and soil data.
(1) Based on the altitude (h) and the ambient temperature (T), the ambient pressure is determined via the ideal gas law.
(2) Soil relative saturation degree ($\tilde{\theta}$) is calculated using the soil moisture (θ) at the topsoil layer and the porosity (ϕ), $\tilde{\theta} = \theta/\phi$.
(3): The dissolved oxygen diffusion coefficient (D_{O_2} [cm^2 s^{-1}]) in water is evaluated using an empirical relation with the ambient temperature (T) [9]: $\log_{10}(D_{\mathrm{O}_2}) = -4.41 + (773.8/T) - (506/T)^2$.
(4) Dissolved oxygen concentration is calculated using the ambient pressure (1) and Henry's law solubility parameter (kH [mol kg^{-1} bar^{-1}]). [10].
(5) Within the Michaelis–Menten kinetic [11, 12], $V_m \frac{C_s}{C_s+K_{m(s)}} \frac{C_{O_2}}{C_{O_2}+K_{m(O_2)}}$, the value of C_s is evaluated based on a dissolved organic database [13]. The maximum reaction rate is given by $V_m = \alpha_s \exp(-E_a/(RT))$; where R is the universal gas constant, T is the surface temperature, and α_s and E_a are the pre-exponential factor and activation energy of the enzymatic reaction, respectively. In the model, the value of the pre-exponential factor, α_s, is proportional to the soil surface area (SSA). Therefore, V_m, in the upscaled model, is a function of the surface temperature and the soil texture (i.e., surface area).
(6) The water patch characteristic properties, namely number (N_c, Eq. 3) and size (S_c, Eq. 3; main text) are evaluated based on the characteristic grain size (λ_c) and the soil saturation degree ($\tilde{\theta}$).
(7) By combining the results from (3), (5) and (6), Eq. 1 can be solved (see main text).
(8) Based on the water patch properties in the system, Eqs. 2 and 3 can be solved to characterize the spatial distribution of water at different saturation conditions.
(9) By multiplying the solution of (7), i.e., the heterotrophic respiration rate for a single water patch, by the number of patches in the domain (8), we can obtain the heterotrophic respiration rate from the soil matrix (of length scale L).

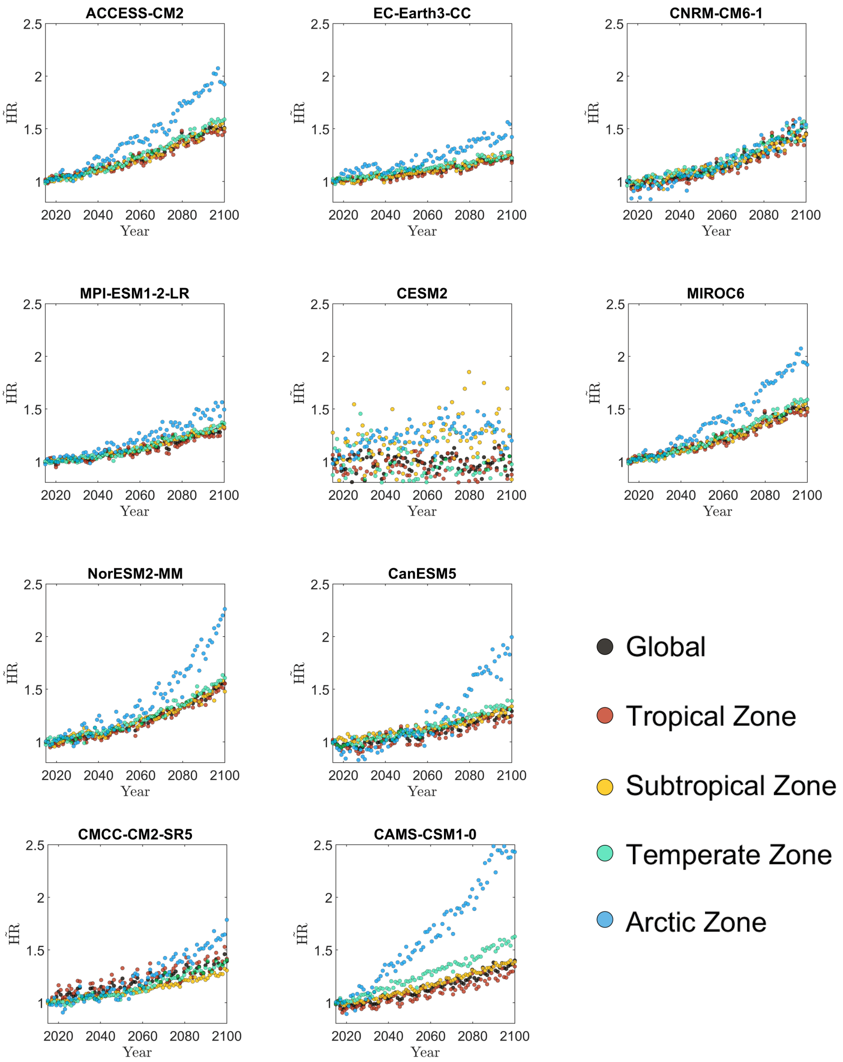

Supplementary Fig.. 7: Projected changes in soil heterotrophic respiration ($\widetilde{HR}$, ratio relative to the reference year of 2015) through the 21st century estimated using climate projections from 10 different climate models. Symbol colours represent different climate zones.

Supplementary Table 2: **Parameters for the two-dimensional pore-scale simulations of Fig. 2a**.

Parameters that depended on temperature (i.e, diffusion coefficients (D_i), Henry's constant (kH) and maximum reaction rate (V_m)) were calculated based on 25 °C.

Parameter	Units	Value	Description	Source
$D_{O_2(g)}$	m^2 s^{-1}	1.9×10^{-5}	Atmospheric oxygen diffusion coefficient	[14]
$D_{CO_2(g)}$	m^2 s^{-1}	1.4×10^{-5}	Atmospheric carbon dioxide diffusion coefficient	[14]
$D_{O_2(aq)}$	m^2 s^{-1}	2.5×10^{-9}	Dissolved oxygen diffusion coefficient	[14]
$D_{CO_2(aq)}$	m^2 s^{-1}	2.1×10^{-9}	Dissolved carbon dioxide diffusion coefficient	[14]
$D_{DOC(aq)}$	m^2 s^{-1}	4.5×10^{-10}	Dissolved organic carbon diffusion coefficient	[15]
kH	mol L^{-1} atm^{-1}	0.0013	Henry's law constant	
$C0_{O_2(g)}$	mol m^{-3}	10	Representative atmospheric oxygen concentration	
$C0_{DOC}$	mg L^{-1}	30 $\pm$(10)	Dissolved organic carbon concentration at the soil grain perimeter	[13]
V_m	mol m^{-2} s^{-1}	6×10^{-12}	Maximum (surface) reaction rate	[12, 16]
$K_{m(s)}$	mol m^{-3}	1 $\pm$(0.025)	Michaelis constant for dissolved organic carbon	[12, 16]
$K_m(O_2)$	mol m^{-3}	0.1 $\pm$(0.05)	Michaelis constant for oxygen	[17]

References

[1] Franzluebbers, A.J.: Microbial activity in response to water-filled pore space of variably eroded southern piedmont soils. Applied Soil Ecology **11**(1), 91–101 (1999). https://doi.org/10.1016/S0929-1393(98)00128-0

[2] Zhang, Y., Schaap, M.G.: Weighted recalibration of the rosetta pedotransfer model with improved estimates of hydraulic parameter distributions and summary statistics (rosetta3). Journal of Hydrology **547**, 39–53 (2017). https://doi.org/10.1016/j.jhydrol.2017.01.004

[3] Yan, D., Li, J., Pei, J., Cui, J., Nie, M., Fang, C.: The temperature sensitivity of soil organic carbon decomposition is greater in subsoil than in topsoil during laboratory incubation. Scientific Reports **7**(1), 5181 (2017). https://doi.org/10.1038/s41598-017-05293-1

[4] Lu, H., Liu, S., Wang, H., Luan, J., Schindlbacher, A., Liu, Y., Wang, Y.: Experimental throughfall reduction barely affects soil carbon dynamics in a warm-temperate oak forest, central china. Scientific Reports **7**(1), 15099 (2017). https://doi.org/10.1038/s41598-017-15157-3

[5] Wu, C., Liang, N., Sha, L., Xu, X., Zhang, Y., Lu, H., Song, L., Song, Q., Xie, Y.: Heterotrophic respiration does not acclimate to continuous warming in a subtropical forest. Scientific Reports **6**(1), 21561 (2016). https://doi.org/10.1038/srep21561

[6] Jian, J., Vargas, R., Anderson-Teixeira, K., Stell, E., Herrmann, V., Horn, M., Kholod, N., Manzon, J., Marchesi, R., Paredes, D., Bond-Lamberty, B.: A restructured and updated global soil respiration database (srdb-v5). Earth System Science Data **13**(2), 255–267 (2021). https://doi.org/10.5194/essd-13-255-2021

[7] Rodell, M., Houser, P.R., Jambor, U., Gottschalck, J., Mitchell, K., Meng, C.-J., Arsenault, K., Cosgrove, B., Radakovich, J., Bosilovich, M., Entin, J.K., Walker, J.P., Lohmann, D., Toll, D.: The global land data assimilation system. Bulletin of the American Meteorological Society **85**(3), 381–394 (2004). https://doi.org/10.1175/BAMS-85-3-381

[8] Todd-Brown, K.E.O., Randerson, J.T., Hopkins, F., Arora, V., Hajima, T., Jones, C., Shevliakova, E., Tjiputra, J., Volodin, E., Wu, T., Zhang, Q., Allison, S.D.: Changes in soil organic carbon storage predicted by earth system models during the 21st century. Biogeosciences **11**(8), 2341–2356 (2014). https://doi.org/10.5194/bg-11-2341-2014

[9] Han, P., Bartels, D.M.: Temperature dependence of oxygen diffusion in H_2O and D_2O. The Journal of Physical Chemistry **100**(13), 5597–5602 (1996). https://doi.org/10.1021/jp952903y

[10] Linstrom, P.: NIST Chemistry WebBook, NIST Standard Reference Database 69. National Institute of Standards and Technology. Type: dataset (1997). https://doi.org/10.18434/T4D303

[11] Davidson, E.A., Samanta, S., Caramori, S.S., Savage, K.: The dual arrhenius and Michaelis–Menten kinetics model for decomposition of soil organic matter at hourly to seasonal time scales. Global Change Biology **18**(1), 371–384 (2012). https://doi.org/10.1111/j.1365-2486.2011.02546.x

[12] German, D.P., Marcelo, K.R.B., Stone, M.M., Allison, S.D.: The Michaelis–Menten kinetics of soil extracellular enzymes in response to temperature: a cross-latitudinal study. Global Change Biology **18**(4), 1468–1479 (2012). https://doi.org/10.1111/j.1365-2486.2011.02615.x

[13] Langeveld, J., Bouwman, A.F., van Hoek, W.J., Vilmin, L., Beusen, A.H.W., Mogollón, J.M., Middelburg, J.J.: Estimating dissolved carbon concentrations in global soils: a global database and model. SN Applied Sciences **2**(10), 1626 (2020). https://doi.org/10.1007/s42452-020-03290-0

[14] Hou, C., Gheorghiu, S., Huxley, V.H., Pfeifer, P.: Reverse engineering of oxygen transport in the lung: Adaptation to changing demands and resources through space-filling networks. PLOS Computational Biology **6**(8), 1–12 (2010). https://doi.org/10.1371/journal.pcbi.1000902

[15] Balch, J., Guéguen, C.: Effects of molecular weight on the diffusion coefficient of aquatic dissolved organic matter and humic substances. Chemosphere **119**, 498–503 (2015). https://doi.org/10.1016/j.chemosphere.2014.07.013

[16] Eberwein, J., Shen, W., Jenerette, G.D.: Michaelis-Menten kinetics of soil respiration feedbacks to nitrogen deposition and climate change in subtropical forests. Scientific Reports **7**(1), 1752 (2017). https://doi.org/10.1038/s41598-017-01941-8

[17] Magliaro, C., Mattei, G., Iacoangeli, F., Corti, A., Piemonte, V., Ahluwalia, A.: Oxygen consumption characteristics in 3D constructs depend on cell density. Frontiers in Bioengineering and Biotechnology **7** (2019). https://doi.org/10.3389/fbioe.2019.00251
