# Supplementary material for: Global warming accelerates soil heterotrophic respiration
Source: Nat Commun. 2023 Jun 10;14:3452. doi: 10.1038/s41467-023-38981-w (PMC10257684; doi:10.1038/s41467-023-38981-w)
Supplement: Supplementary file 3 — Reporting Summary [file 41467_2023_38981_MOESM3_ESM.pdf]

nature portfolio

Corresponding author(s): Alon Nissan

Last updated by author(s): May 17, 2023

Reporting Summary

Nature Portfolio wishes to improve the reproducibility of the work that we publish. This form provides structure for consistency and transparency in reporting. For further information on Nature Portfolio policies, see our Editorial Policies and the Editorial Policy Checklist.

Statistics

For all statistical analyses, confirm that the following items are present in the figure legend, table legend, main text, or Methods section.

n/a	Confirmed	
x		The exact sample size (*n*) for each experimental group/condition, given as a discrete number and unit of measurement
x		A statement on whether measurements were taken from distinct samples or whether the same sample was measured repeatedly
x		The statistical test(s) used AND whether they are one- or two-sided<br>*Only common tests should be described solely by name; describe more complex techniques in the Methods section.*
x		A description of all covariates tested
x		A description of any assumptions or corrections, such as tests of normality and adjustment for multiple comparisons
	x	A full description of the statistical parameters including central tendency (e.g. means) or other basic estimates (e.g. regression coefficient) AND variation (e.g. standard deviation) or associated estimates of uncertainty (e.g. confidence intervals)
x		For null hypothesis testing, the test statistic (e.g. *F*, *t*, *r*) with confidence intervals, effect sizes, degrees of freedom and *P* value noted<br>*Give P values as exact values whenever suitable.*
x		For Bayesian analysis, information on the choice of priors and Markov chain Monte Carlo settings
x		For hierarchical and complex designs, identification of the appropriate level for tests and full reporting of outcomes
	x	Estimates of effect sizes (e.g. Cohen's *d*, Pearson's *r*), indicating how they were calculated

Our web collection on statistics for biologists contains articles on many of the points above.

Software and code

Policy information about availability of computer code

Data collection	No software was used in data collection. All data used was publicly available in an easy-to-download format.
Data analysis	All data and code are available on the Gitlab's website as stated in the manuscript; https://gitlab.ethz.ch/anissan/global-warming-accelerates-soil-heterotrophic-respiration

For manuscripts utilizing custom algorithms or software that are central to the research but not yet described in published literature, software must be made available to editors and reviewers. We strongly encourage code deposition in a community repository (e.g. GitHub). See the Nature Portfolio guidelines for submitting code & software for further information.

Data

Policy information about availability of data

All manuscripts must include a data availability statement. This statement should provide the following information, where applicable:

- Accession codes, unique identifiers, or web links for publicly available datasets
- A description of any restrictions on data availability
- For clinical datasets or third party data, please ensure that the statement adheres to our policy

Before publication all raw data are archived on Zenodo; https://zenodo.org/record/7918484#.ZFyxnOxBwrk

Human research participants

Policy information about studies involving human research participants and Sex and Gender in Research.

Reporting on sex and gender	n/a
Population characteristics	n/a
Recruitment	n/a
Ethics oversight	n/a

Note that full information on the approval of the study protocol must also be provided in the manuscript.

Field-specific reporting

Please select the one below that is the best fit for your research. If you are not sure, read the appropriate sections before making your selection.

☐ Life sciences ☐ Behavioural & social sciences ☒ Ecological, evolutionary & environmental sciences

For a reference copy of the document with all sections, see nature.com/documents/nr-reporting-summary-flat.pdf

Ecological, evolutionary & environmental sciences study design

All studies must disclose on these points even when the disclosure is negative.

Study description	We formulated a mathematical framework for soil heterotrophic respiration based on pore scale (soil) biophysical mechanisms and upscaled it to account for ecosystem environmental forcings at coarser scales. Numerical simulations, laboratory measurements, and field observations allow us to validate the new approach from pore to global scale. Finally, we use the model to predict the evolution of soil heterotrophic respiration based on projections of future climate change derived from the most recent CMIP6 climate change experiments.
Research sample	The primary datasets we used existed on a global scale and are freely available to the public (see our manuscript for more details about the datasets).
Sampling strategy	We did not perform any sampling for this analysis. Instead, we chose (mainly) NASA's GLDAS and CMIP6 ensemble datasets to calibrate our model and make simulations with it for a future climate. We chose these datasets because of their reliability and widespread use in the scientific community.
Data collection	No data collection was needed; all data was freely and publicly available.
Timing and spatial scale	Data was downloaded in the late of 2021.
Data exclusions	No data was excluded
Reproducibility	The results are fully reproducible with the data and methods provided.
Randomization	n/a
Blinding	n/a

Did the study involve field work? ☐ Yes ☒ No

Reporting for specific materials, systems and methods

We require information from authors about some types of materials, experimental systems and methods used in many studies. Here, indicate whether each material, system or method listed is relevant to your study. If you are not sure if a list item applies to your research, read the appropriate section before selecting a response.

Materials & experimental systems

n/a	Involved in the study
☒	☐ Antibodies
☒	☐ Eukaryotic cell lines
☐	☐ Palaeontology and archaeology
☒	☐ Animals and other organisms
☒	☐ Clinical data
☒	☐ Dual use research of concern

Methods

n/a	Involved in the study
☒	☐ ChIP-seq
☒	☐ Flow cytometry
☒	☐ MRI-based neuroimaging

Palaeontology and Archaeology

Specimen provenance: n/a

Specimen deposition: n/a

Dating methods: n/a

☐ Tick this box to confirm that the raw and calibrated dates are available in the paper or in Supplementary Information.

Ethics oversight: n/a

Note that full information on the approval of the study protocol must also be provided in the manuscript.
